# Multiomics: Two-Sample, Bidirectional, Multivariate and Mediated Mendelian Randomization Analysis of Allergic Rhinitis

**DOI:** 10.1007/s12070-025-05413-8

**Published:** 2025-03-17

**Authors:** Cheng Zhong, Li-hua Wang, Hao-peng Zhang, Lin Ji, Yu Guo

**Affiliations:** 1 Shanghai Municipal Hospital of Traditional Chinese Medicine, Shanghai University of Traditional Chinese Medicine, Shanghai, China; 2 Department of Otolaryngology, Shanghai Municipal Hospital of Traditional Chinese Medicine, Shanghai University of Traditional Chinese Medicine, Shanghai, China

**Keywords:** Multiomics, Mendelian randomization, Allergic rhinitis, Causal relationships

## Abstract

This study investigates the potential causal relationships among immune cells, plasma liposomes, and plasma metabolites in allergic rhinitis (AR) using a two-sample bidirectional multivariate and mediated Mendelian Randomization (MR) approach. Genetic data from five GWAS datasets covering traits linked to immune cells, plasma liposomes, plasma metabolites, and AR were used. Instrumental variables were selected based on strict criteria to ensure independence and exclude confounders. The primary MR analysis employed the Inverse Variance Weighted method, supplemented by sensitivity analyses using the Weighted Median and MR-Egger methods. The MR analyses suggest a genetic correlation among immune cells, plasma liposomes, and plasma metabolites, influencing the risk of AR. Findings were robust across various sensitivity analyses, supporting the absence of pleiotropy and outliers. No reverse causality was found, indicating a unidirectional influence. Genetic predispositions to immune cells, plasma liposomes, and plasma metabolites significantly change the risk of developing AR. Further studies are needed to explore these associations in diverse populations and refine the underlying mechanisms.

**Supplementary Information:**

The online version contains supplementary material available at 10.1007/s12070-025-05413-8.

## Introduction

Allergic rhinitis (AR) is an IgE-mediated, noninfectious inflammation of the nasal mucosa caused by inhaled allergens in atopic individuals, involving various immune cells and cytokines [1]. Symptoms include nasal obstruction, rhinorrhea, sneezing, and nasal itching.^1^ AR prevalence is rising globally, especially in low- and middle-income countries, with rates ranging from 1.0–54.5%. [2–3]

The mechanisms of AR are not well understood. Multiple immune cells are involved in AR development, and liposomes can act as antagonists. Intranasal ceramide liposomes reduce sneezing and mast cell and eosinophil degranulation in nasal tissue [4]. Further study is needed to determine if plasma liposomes and metabolites work synergistically with immune cells in AR.

Mendelian randomization (MR) is used to explore causal relationships between exposures and outcomes, employing single nucleotide polymorphisms (SNPs) as proxies to bypass confounding and reverse causality issues. Regulatory T cells are reduced in AR patients, resulting in immune dysregulation. This study primarily explored the genetic-level causal relationship between immune cells and liposomes in AR using univariable, bidirectional, and multivariable MR analyses.

## Materials and Methods

### Study Design

**Fig. 1:**
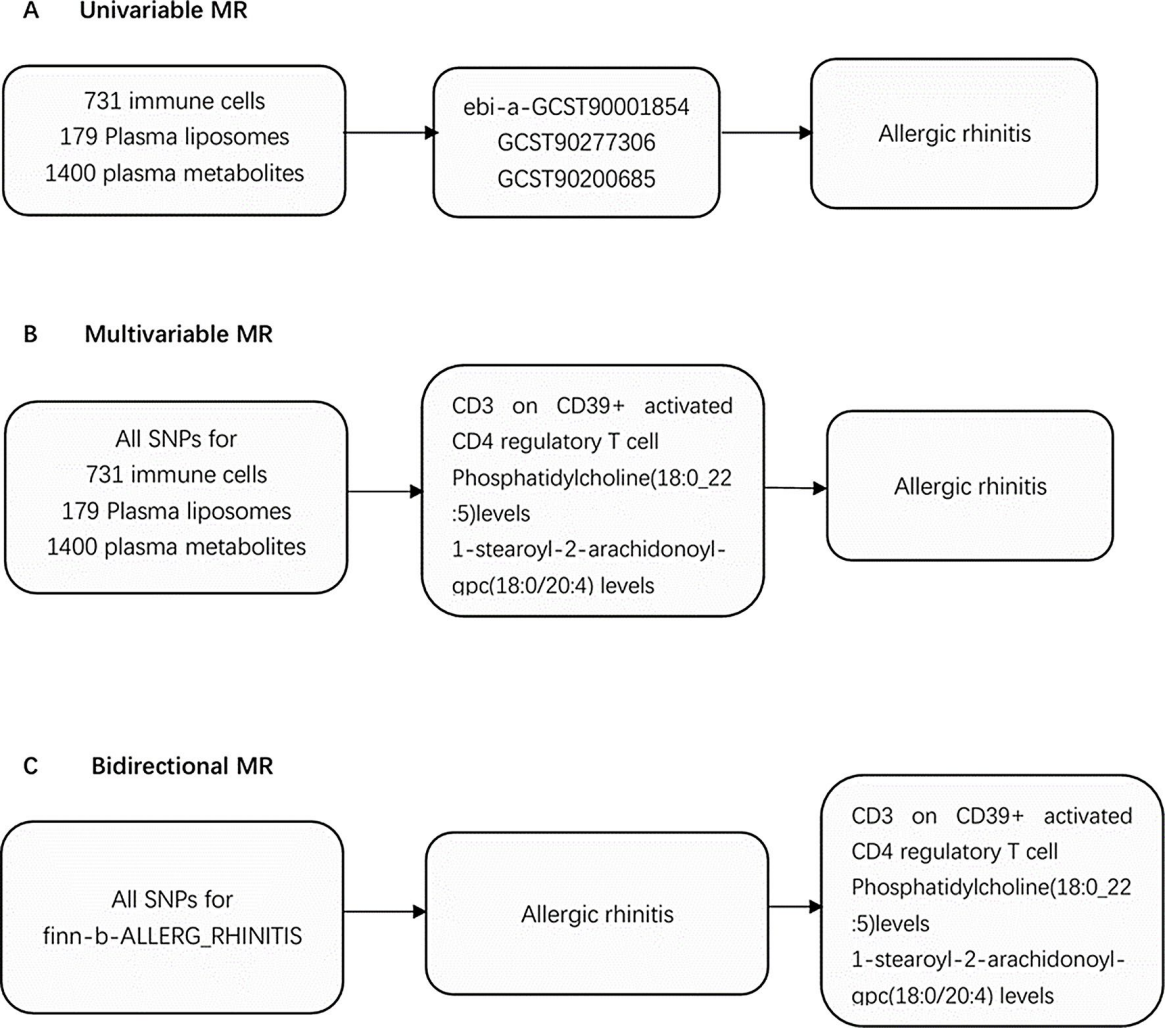
Schematic presentation of (**A**) univariable, (**B**) multivariable, and (**C**) bidirectional

To explore the causal relationship among immune cells, plasma liposomes, plasma metabolites, and AR, we performed a univariable MR analysis (Fig. 1A). Genetic variations used as instrumental variables (IVs) met three core assumptions: (1) IVs were significantly associated with the exposure, (2) IVs were unrelated to confounding factors, and (3) IVs influenced outcomes only through their effect on the exposure [5]. To reduce the likelihood of reverse causation, we conducted a bidirectional MR analysis (Fig. 1B) and a multivariable MR analysis to evaluate the direct impact of immune cells on AR (Fig. 1C).

### GWAS Data Source

The genome-wide association study (GWAS) data on immune cells were obtained from a recent study involving 3,757 individuals of Sardinian descent within the European population.6 This study included 3,757 cases and 3,027 controls, with participants aged 18 to 102 years, and a gender distribution of 43% male and 57% female.6 The study covered 731 immunophenotypes, including 118 absolute cell (AC) counts, 389 median fluorescence intensities (MFIs), 32 morphological parameters (MPs), and 192 relative cell (RC) counts.6 The study measured 22 million genetic variations [6]. We performed MR analysis using the GWAS IDs (GCST90001391-GCST90002121).

GWAS data on plasma lipidome came from a study involving 7,174 individuals of Sardinian descent within the European population.7 This study included 179 immunophenotypes across 13 lipid types [7]. We used the GWAS IDs (GCST90277238-GCST90277416) provided by the authors for MR analysis.

Summary statistics linking the human metabolome with genetic variation were derived from a large-scale GWAS involving 1,091 metabolites and 309 metabolite ratios in 8,299 individuals from the Canadian Longitudinal Study on Aging (CLSA) cohort [8]. Public databases of these metabolites are available from the GWAS Catalog (https://www.ebi.ac.uk/gwas/), with accession numbers for European GWASs: GCST90199621-90201020, and for non-European GWASs: GCST90201021-90204063.

AR data were sourced from the FinnGen database (https://www.finngen.fi/fi). This dataset includes 12,240 cases, 392,069 controls, and 21,306,212 single-nucleotide polymorphisms (SNPs). AR diagnoses were made according to the International Classification of Diseases, specifically ICD-10 (J30.4) coding standards. All populations included were of European origin. Additional information is available from the FinnGen website (Risteys · Home (finregistry.fi)).

Our job is to perform a secondary analysis of the data, which does not require an ethical audit. The original data has already undergone an ethics review, and we are doing a secondary analysis on the original data, so there is no need to apply for additional ethics.

### Selection of Instrumental Variables

We implemented stringent quality controls to select IVs that meet the three core assumptions of MR analysis, ensuring robustness and reliability. First, we chose SNPs of immune cells at a genome-wide significance threshold (*p* < 1e − 05) [9]. We addressed linkage disequilibrium (LD) by removing highly linked variants (r2 = 0.001, clumping distance of 10,000 kb) to minimize potential bias.^9^ We used the PhenoScanner database to exclude confounding factors. Finally, we calculated F-statistics for all selected SNPs, excluding those with F-statistics less than [10]to ensure strong associations with the exposure [10]. The F-statistics were computed using the formula R2 = 2*(1-MAF)MAFbeta^2; F = R2/(1-R)2(N-2) [11, 12]. (Supplementary Table 1).

### Statistical Analysis

In our MR analysis, we used the inverse-variance weighted (IVW) method as the primary analytical approach. Statistical significance was determined with p-value thresholds set at 6.84E − 05/2.79E-04, adjusted using the Bonferroni method (0.05/731) for immune cells and (0.05/179) for plasma lipidome. Given the large number of plasma metabolite classes, we applied the Benjamini-Hochberg method to correct for the 1400 plasma metabolomes (fdr < 0.05).

Since the IVW method assumes no intercept term, we performed the MR-Egger test to check for the presence of an intercept [13]. Additionally, we used the MR-Egger, weighted median, weighted mode, and simple mode methods to enhance result robustness.

We conducted Cochran’s Q test and MR-Egger intercept analysis to ensure no heterogeneity and pleiotropy. Heterogeneity was considered present if the Q–p-value was less than 0.05, prompting the use of a random effects model for analysis [14]. An MR-Egger intercept p-value above 0.05 indicated the absence of pleiotropy [15]. We employed leave-one-out analysis to assess the potential impact of single SNPs on the causal relationship between immune cells and AR.

All statistical analyses were performed using the “TwoSampleMR” (version 0.5.7) packages within R statistical software (version 4.3.3). Additionally, we conducted multivariate Mendelian randomized analysis of both immune cells and liposomes for AR using the MVMR package within R (version 4.3.3). To prevent individual SNPs from unduly influencing the causal relationship, we applied the “leave-one-out” method to exclude abnormal SNPs. Results were visually represented through forest plots, funnel plots, scatter plots, and “leave-one-out” plots.

## Result

### Univariable MR

**Fig. 2:**
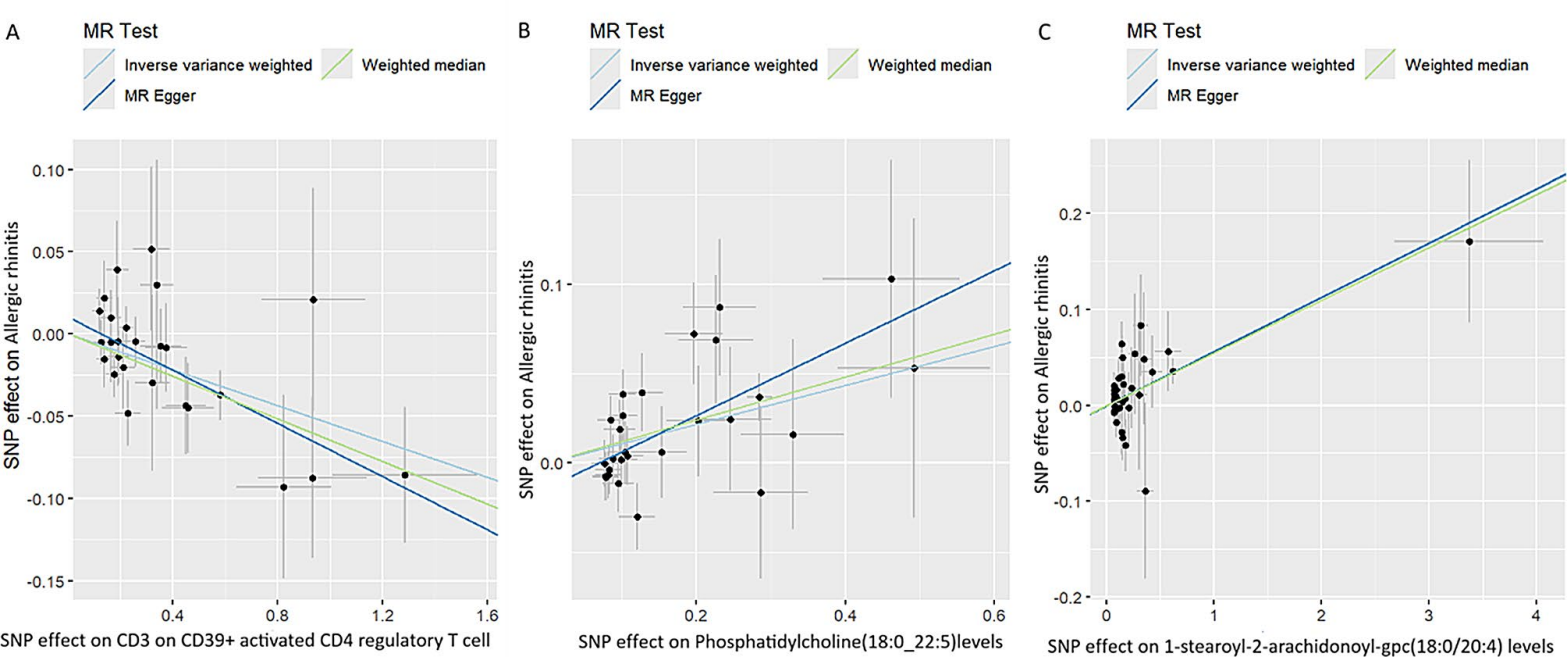
Scatter plots of effects of immune cells, plasma liposomes and plasma metabolites on the AR using univariable MR. (**A**) CD3 on CD39 + activated CD4 regulatory T cell. (**B**) Phosphatidylcholine(18:0_22:5)levels. (**C**) 1-stearoyl-2-arachidonoyl-gpc(18:0/20:4) levels

**Fig. 3:**
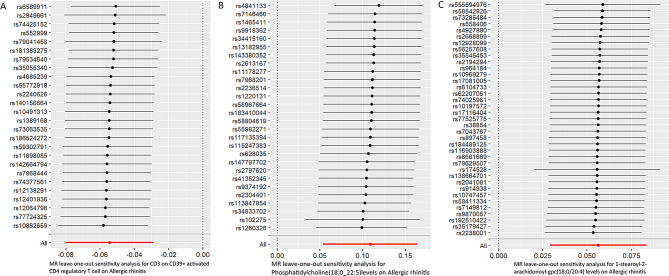
MR leave-one-out sensitivity analysis of immune cells, plasma liposomes and plasma metabolites on the AR using univariable MR. (**A**) CD3 on CD39 + activated CD4 regulatory T cell. (**B**) Phosphatidylcholine(18:0_22:5)levels. (**C**) 1-stearoyl-2-arachidonoyl-gpc(18:0/20:4) levels

We examined the relationship between 731 immune cells and AR (Supplementary Table 2). Our analysis identified that 56 of these immune cells had significant associations with AR, including 35 risk factors and 21 protective factors (Supplementary Table 3). However, we excluded 55 immune cell influencing factors because they were not corrected by Bonferroni, potentially leading to false positives. Consequently, we identified one potential protective factor. We calculated terminally differentiated CD3 on CD39 + activated CD4 regulatory T cells using a random effects model (Table 1). After applying the Bonferroni test, we identified one protective factor associated with AR (Table 1; Figs. 2 and 3). CD3 on CD39 + activated CD4 regulatory T cells was found to significantly reduce the risk of AR (p: 3.0594e-05, OR: 0.94699, 95%CI: 0.92305–0.97155).

We then investigated the association between plasma lipidome and AR (Supplementary Table 4). Our analysis revealed that 39 plasma lipidomes were significantly associated with AR, comprising 8 risk factors and 31 protective factors (Supplementary Table 5). However, we excluded 38 plasma lipidome influencing factors because they were not corrected by Bonferroni, potentially leading to false positives. Consequently, we identified one potential risk factor. We calculated terminally differentiated Phosphatidylcholine (18:0_22:5) levels using a random effects model (Table 1). After applying the Bonferroni test, we identified one risk factor associated with AR (Table 1; Figs. 2 and 3). Phosphatidylcholine (18:0_22:5) levels were found to significantly decrease the risk of AR (p: 1.09337e-04, OR: 1.11516, 95% CI: 1.05525–1.17846).

Additionally, we explored the relationship between the plasma metabolome and AR (Supplementary Table 6). Our analysis showed that 86 plasma lipidomes had significant associations with AR, including 43 risk factors and 43 protective factors (Supplementary Table 7). However, we excluded 85 plasma metabolome influencing factors because they were not corrected by Benjamini-Hochberg, potentially leading to false positives. Consequently, we identified one potential risk factor. We calculated terminally differentiated 1-stearoyl-2-arachidonoyl-gpc (18:0/20:4) levels using a random effects model (Table 1). Following the Bonferroni test, we identified one risk factor associated with AR (Table 1; Figs. 2 and 3). 1-stearoyl-2-arachidonoyl-gpc (18:0/20:4) levels could significantly increase the risk of AR (p: 5.12306e-05, OR: 1.05788, 95%CI: 1.02946–1.08708).

**Table 1:** The result of the univariable and the reverse MR

Exposure	Outcome	Method	nSNP	pval	OR	95%CI
CD3 on CD39 + activated CD4 regulatory T cell	allergic rhinitis	IVW: random effects	26	3.11570e-05	0.94704	0.92310–0.97161
Phosphatidylcholine(18:0_22:5)levels	allergic rhinitis	IVW: random effects	28	1.09337e-04	1.11516	1.05525–1.17846
1-stearoyl-2-arachidonoyl-gpc(18:0/20:4) levels	allergic rhinitis	IVW: random effects	37	5.12306e-05	1.05788	1.02946–1.08708
allergic rhinitis	CD3 on CD39 + activated CD4 regulatory T cell	IVW: random effects	57	0.3229288	0.95209	0.86378–1.04943
allergic rhinitis	Phosphatidylcholine(18:0_22:5)levels	IVW: random effects	59	0.4514637	0.97516	0.91337–1.04114
allergic rhinitis	1-stearoyl-2-arachidonoyl-gpc(18:0/20:4) levels	IVW: random effects	62	0.4218475	1.02147	0.96987–1.07582

IVW, inverse-variance weighted; OR, odds ratio; CI, confidence interval; MR, Mendelian randomization

### Bidirectional MR

To demonstrate whether AR has an inverse causal relationship with immune cells, plasma liposomes, and plasma metabolites, we performed a two-way Mendelian randomization analysis. No significant reverse causality was found (*P* > 0.05) (Table 1).

### Multivariable MR

We performed multivariate MR of corrected CD3 on CD39 + activated CD4 regulatory T cells, Phosphatidylcholine (18:0_22:5) levels, and 1-stearoyl-2-arachidonoyl-gpc (18:0/20:4) levels. Multivariate MR revealed that CD3 on CD39 + activated CD4 regulatory T cells could decrease AR risk (p: 0.002, Estimate: -0.043, 95%CI: -0.070 – -0.016), while Phosphatidylcholine (18:0_22:5) levels could increase AR risk (*p* < 0.001, Estimate: 0.110, 95%CI: 0.058 – 0.162) (Table 2). The condition F statistics for the strength of the instrument are listed in the table.

In multivariable MR analysis including CD3 on CD39 + activated CD4 regulatory T cells and 1-stearoyl-2-arachidonoyl-gpc (18:0/20:4) levels, CD3 on CD39 + activated CD4 regulatory T cells could decrease AR risk (*p* < 0.001, Estimate: -0.055, 95%CI: -0.080 – -0.030), while 1-stearoyl-2-arachidonoyl-gpc (18:0/20:4) levels could increase AR risk (*p* < 0.001, Estimate: 0.056, 95%CI: 0.029 – 0.083) (Table 3).

**Table 2:** The result of the multivariable MR of immune cells and plasma liposomes

Exposure	Outcome	Method	Estimate	Std Error	95%CI	pval	F
CD3 on CD39 + activated CD4 regulatory T cell	allergic rhinitis	IVW: random effects	-0.043	0.014	-0.070- -0.016	0.002	22.57768
Phosphatidylcholine(18:0_22:5)levels	allergic rhinitis	IVW: random effects	0.110	0.027	0.058–0.162	<0.001	18.06723

IVW, inverse-variance weighted; CI, confidence interval; MR, Mendelian randomization

**Table 3:** The result of the multivariable MR of immune cells and plasma metabolites

Exposure	Outcome	Method	Estimate	Std Error	95%CI	pval	F
CD3 on CD39 + activated CD4 regulatory T cell	allergic rhinitis	IVW: random effects	-0.055	0.013	-0.080- -0.030	<0.001	18.75388
1-stearoyl-2-arachidonoyl-gpc(18:0/20:4) levels	allergic rhinitis	IVW: random effects	0.056	0.014	0.029- 0.083	<0.001	43.74498

IVW, inverse-variance weighted; CI, confidence interval; MR, Mendelian randomization

### Mediator Mendelian Randomization

We further performed mediated MR analysis to explore the regulatory role of immune cells on plasma liposomes and plasma metabolites. However, no significant intermediary relationship was found (*P* > 0.05) (Table 4).

**Table 4:** The result of the mediating Mendelian randomization

Exposure	Outcome	Method	nSNP	pval	OR	95%CI
CD3 on CD39 + activated CD4 regulatory T cell	Phosphatidylcholine(18:0_22:5)levels	IVW: random effects	23	0.7050135	0.99324	0.95899–1.02872
Phosphatidylcholine(18:0_22:5)levels	CD3 on CD39 + activated CD4 regulatory T cell	IVW: random effects	27	0.7846138	0.98050	0.85139–1.12920
CD3 on CD39 + activated CD4 regulatory T cell	1-stearoyl-2-arachidonoyl-gpc(18:0/20:4) levels	IVW: random effects	25	0.7028492	0.99415	0.96466–1.02456
1-stearoyl-2-arachidonoyl-gpc(18:0/20:4) levels	CD3 on CD39 + activated CD4 regulatory T cell	IVW: random effects	none	---	---	---

### Sensitivity Analyses

We conducted Cochran’s Q test and MR-Egger intercept test to assess the robustness of our results. The MR-Egger intercept test p-values for Phosphatidylcholine (18:0_22:5) levels, CD3 on CD39 + activated CD4 regulatory T cells, and 1-stearoyl-2-arachidonoyl-gpc (18:0/20:4) levels were all greater than 0.05, indicating no horizontal pleiotropy (Table 5). No anomalies were identified during the leave-one-out test.

The p-values of Cochran’s Q test were larger than 0.05 for 1-stearoyl-2-arachidonoyl-gpc (18:0/20:4) levels, CD3 on CD39 + activated CD4 regulatory T cells, and Phosphatidylcholine (18:0_22:5) levels, indicating no heterogeneity (Table 6). Consequently, we used the random effects model to analyze the effects on AR (Table 1; Figs. 2 and 3). Our results have been validated and are reliable.

**Table 5:** The result of the horizontal pleiotropy results

Exposure	egger_intercept	pval
CD3 on CD39 + activated CD4 regulatory T cell	0.01008694	0.160269
Phosphatidylcholine(18:0_22:5)levels	-0.01383678	0.07015338
1-stearoyl-2-arachidonoyl-gpc(18:0/20:4) levels	-8.898198e-05	0.9824234

**Table 6:** The result of the heterogeneity results

Exposure	Q	Q_pval
CD3 on CD39 + activated CD4 regulatory T cell	21.22087	0.6802312
Phosphatidylcholine(18:0_22:5)levels	32.48272	0.2146779
1-stearoyl-2-arachidonoyl-gpc(18:0/20:4) levels	32.27453	0.6464734

## Discussion

AR is a nasal inflammation caused by complex etiologies, making its diagnosis and management challenging. AR can be classified into MAR and MSAR based on clinical symptom scores, but this method is subjective and relatively insensitive. Previous studies proposed various indicators to evaluate AR severity, such as nasal nitric oxide, lipids, peripheral lymphocytes, and metabolites, but none demonstrated adequate sensitivity and specificity. Thus, identifying objective biomarkers for AR diagnosis and accurately reflecting disease severity remains a significant research area.

In this study, we used univariable and multivariable MR to investigate associations among 731 immune cells, 179 plasma lipidomes, 1400 plasma metabolomes, and AR. Using extensive public GWAS data, we uncovered complex relationships between immune cells, plasma lipidomes, plasma metabolomes, and AR.

Our findings were confirmed by sensitivity analyses. We applied the Bonferroni test to support the association between immune cells and AR, and a similar approach should validate the association between plasma lipidomes and AR. The Benjamini-Hochberg method supported the association between plasma metabolomes and AR.

Through univariable MR combined with the Bonferroni test, we identified one risk factor and one protective factor. Phosphatidylcholine (18:0_22:5) levels increase AR risk through multivariable MR after controlling for immune cells. CD3 on CD39 + activated CD4 regulatory T cells decrease AR risk through multivariable MR after controlling for plasma lipidomes. 1-stearoyl-2-arachidonoyl-gpc (18:0/20:4) levels increase AR risk through multivariable MR after controlling for immune cells. Bidirectional MR did not reveal a bidirectional link between immune cells and AR, nor between plasma lipidomes, plasma metabolites, and AR.

Immune cells can co-regulate AR with plasma liposomes and metabolites, but the co-regulation between plasma liposomes and metabolites is not clear. Multivariate MR and mediated MR analyses did not show significant intermediary relationships, so only bivariate and multivariate results were discussed.

### Univariable MR

Our findings indicate that CD3 on CD39 + activated CD4 regulatory T cells exhibit protective effects on AR. Peripheral multiple cytokine profiles vary significantly in AR patients and are associated with disease severity. Discover-validation cohorts suggest serum CD39 as a novel biomarker for diagnosing AR and reflecting severity [16]. The results analyzed in MR are consistent with this hypothesis. Phosphatidylcholine (18:0_22:5) levels contribute to AR development, while 1-stearoyl-2-arachidonoyl-gpc (18:0/20:4) levels could increase AR risk.

There is strong evidence that high-density lipoprotein (HDL) modulates the immune response, but the role of HDL in allergies is poorly understood. Many AR patients develop a late reaction characterized by monocyte and eosinophil infiltration into the nasal submucosa. HDL dysfunction in AR patients may not suppress inflammation and cellular infiltration. Mass spectrometry and biochemical analysis show reduced levels of apolipoprotein A-I and phosphatidylcholine in AR-HDL. Previous studies indicated that AR is associated with complex alterations in HDL composition and function [17].

### Multivariable MR

We identified potential causative factors of AR through univariable MR. The Bonferroni test reduced type I error probability and increased result stability. However, AR pathogenesis involves various metabolites and biological processes. We performed multivariate MR analysis of three immune cells and metabolites screened by BF and BH tests to explore if multi-omics joint action affects AR development. These methods significantly reduced confounding factors’ influence and increased result confidence. CD3 on CD39 + activated CD4 regulatory T cells can inhibit AR development. Previous studies identified CD39 as a novel biomarker for diagnosing and reflecting AR severity, with levels decreasing in patients [16]. CD39, also known as ectonucleotide triphosphate diphosphohydrolase-1 (E-NTPDase1), is the most prominent ATP hydrolyzing enzyme [18–20]. Extracellular ATP acts as a danger signal in innate immunity, triggering inflammasome activation, oxidative stress response, and promoting proinflammatory cytokines such as IL-1β and IL-8 [21–23]. CD39 catalyzes extracellular ATP and ADP into AMP, promotes adenosine generation, and serves as a critical immunoregulatory molecule that suppresses inflammation [24–26]. Recent studies show gene knockouts of CD39 and CD73 exacerbate allergic airway inflammation in mice by increasing cytokine production and eosinophil recruitment [21, 27]. Another study by Huang et al. reported that CD39 alleviates airway hyperresponsiveness, eosinophilia, mucin deposition, and Th2 cytokine production, acting as a critical regulator in airway inflammation [22]. Further research is needed to elucidate specific mechanisms.

### Strengths and Limitations

Our MR analysis has several strengths. We used a comprehensive approach, including univariable and multivariable MR, to address confounding factors and reverse causality. We conducted multiple sensitivity analyses to validate hypotheses and minimize bias. Our research was limited to the European population to reduce population bias. We used the Bonferroni test to confirm the causative role of AR.

However, our study has limitations. The conclusions cannot be generalized to other populations as our data originated from European sources. The relatively small sample size may introduce bias, highlighting the need for larger samples to ensure robust results.

## Conclusion

Our extensive MR analysis unveiled intricate relationships among plasma lipidome, plasma metabolites, immune cells, and AR. These immune cells can serve as protective factors, while plasma liposomes and plasma metabolites aggravate AR symptoms, opening new perspectives for AR treatment and prevention. Further experiments are necessary to elucidate underlying mechanisms. Our study can guide future research on multiomic analysis of metabolic and biological processes in AR.

## Electronic Supplementary Material

Below is the link to the electronic supplementary material.

Supplementary Material 1

Supplementary Material 2

Supplementary Material 3

Supplementary Material 4

Supplementary Material 5

Supplementary Material 6

Supplementary Material 7

## Data Availability

Publicly available datasets were analyzed in this study. Data can be found at: GWAS MRC-IEU, GWAS Catalog.
